# English to Arabic Translation of the Composite Abuse Scale (CAS): A Multi-Method Approach

**DOI:** 10.1371/journal.pone.0075244

**Published:** 2013-09-25

**Authors:** Samia Alhabib, Gene Feder, Jeremy Horwood

**Affiliations:** 1 Centre for academic primary care, School of Social and Community Medicine, University of Bristol, Bristol, United Kingdom; 2 Bristol Randomised Trials Collaboration, the Centre for Academic Primary Care, NIHR School for Primary Care Research, School of Social and Community Medicine, University of Bristol, Bristol, United Kingdom; University of Pennsylvania, United States of America

## Abstract

**Background:**

The composite abuse scale (CAS) is a comprehensive tool used to measure intimate partner violence (IPV). The aim of the present study is to translate the CAS from English to Arabic.

**Methods:**

The translation of the CAS was conducted in four stages using a multi-method approach: 1) preliminary forward translation, 2) discussion with a panel of bilingual experts, 3) focus groups discussion, and 4) back-translation of the CAS. The discussion included a linguistic validation by a comparison of the Arabic translation with the original English by assessing conceptual and content equivalence.

**Findings:**

In all the stages of translation, there was an agreement to remove the question from the CAS that asked women about the use of objects in the vagina. Wording, format and order of the items were refined according to comments and suggestions made by the experts’ panel and focus groups’ members. The back-translated CAS showed similar wording and language of the original English version.

**Conclusions:**

The Arabic version of the CAS will help to measure the problem of IPV among Saudi women and possibly other Arabic-speaking women in future studies. This is important, particularly, in longitudinal studies or intervention studies among abused women and it allows a comparison of the results of studies from different cultures. However, further validations studies are needed to ensure accurate and equivalent Arabic translation of the CAS.

## Introduction

Translation is an activity of enormous importance in the modern world. It is an art as well as a skill and a science [1]. Catford has identified translation as the replacement of textual material in one language (Source Language=SL) by equivalent textual material in another language (Target Language=TL) [2]. The key term is ‘equivalence’. Equivalence is to replicate the same situation as in the original language, whilst using completely different wording [3].

This paper reports the translation and cultural adaptation of the Composite Abuse Scale (CAS) [4,5] in preparation for a larger study that will be conducted to measure intimate partner violence (IPV) among Saudi women in the UK (Appendix S1 in File S1). IPV has been reported to be prevalent in several Arabic countries [6-16]. However, we focused here in discussing the translation process and not explaining the previous research of IPV among Arabic-speaking countries, which will be discussed in the next paper that we will publish.

The CAS have translated from English into Russian [17], Dutch [18], Turkish and Arabic language [19]. Methods of translation and adaption process were not reported; making it difficult to assess the accuracy of the translated instruments. The consensus of research in the area of translation suggested that translators should be convened to adapt items across languages, conducting forward translation and discussing contestable terms as a group, followed by independent back translation [20]. An accurate translation takes into consideration the dialect of place where the word is used. Moreover, the diversity of people and the geographical distances that separate them lead people to have different linguistic systems. Therefore, language is not only a set of verbal and syntactic forms. It also encodes a peculiar system of ideas and thoughts. This is particularly true when the subject of translation is of a sensitive nature such as intimate partner violence. Hence, one should make an effort to use a general dialect that is trans-cultural and commonly used across Arabic speaking countries.

Accurate translation and appropriate cultural adaptation of a questionnaire allows comparison of findings between populations speaking different languages. Hence, the aim of this study was to translate the composite abuse scale from English to Arabic. The translation of CAS was part of a large project that aimed to investigate the prevalence and experiences of Saudi women living in the UK about their IPV.

## Methods

**Figure 1:**
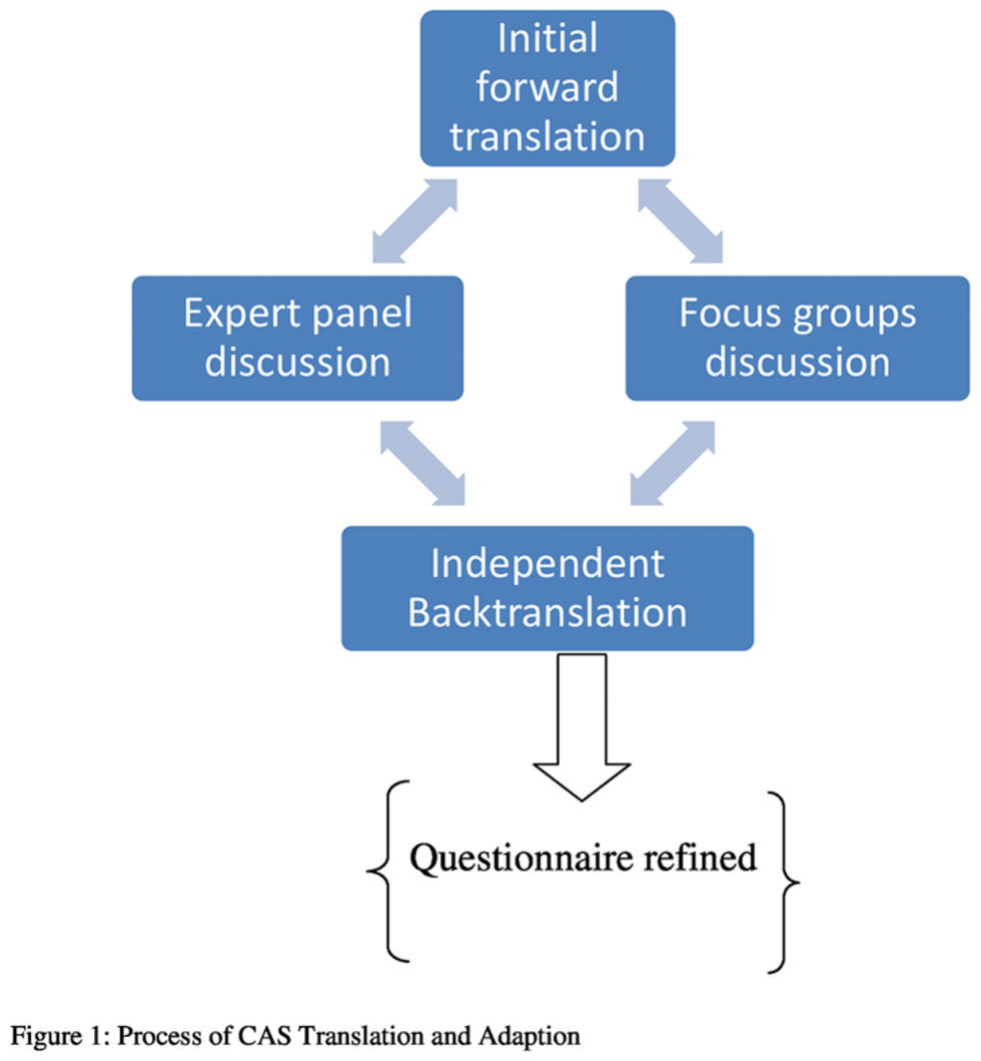
Process of CAS Translation and Adaption.

This study was conducted in the UK. This is because UK is expanding with a growth of multi-cultural population. Also, the researchers live in the UK and were interested to explore IPV among this population and hence; the attempt to translate CAS in preparation of exploring IPV. The translation of the CAS was conducted in four stages (Figure 1) using a multi-method approach. In the first stage, S A conducted a preliminary forward translation. The second stage was carried out through discussion between S A and two panels of bilingual experts: a consultant psychiatrist and a layperson to comment on the initial translation of the CAS. The third stage involved two focus groups discussing the format and wording of the questionnaire. The fourth stage involved the back-translation of the CAS by a professional independent bilingual translator in order to check the accuracy of the original translation. Study methodology was informed and based on International Test Commission (ITC) guidelines [21].

### Forward translation of the CAS

The approach to the translation was in a back and forth manner. S A is a bilingual General Practitioner. She tried not to rely on the precise dictionary translation of each word, i.e. word-for-word process, but relating it to the context of the whole sentence to reflect the equivalent of the English version. She also adopted a process of paraphrasing to translate some sentences in the source text (the English CAS version) in order to get the equivalent meaning in Arabic. This was carried out by referring back to the source text material that was developed in English, and checking comprehension of the source text by referring sometimes to the bilingual Oxford dictionary. After the target text was written in Arabic, every segment was revised by reading and re-reading it, to ensure the replacement of textual material in the English by the equivalent textual material in Arabic.

### Panel translation of CAS

Two bilingual experts reviewed the initial forward translation of the CAS: a bilingual consultant psychiatrist, and a bilingual layperson. Both have experiences in dealing with abused women as described in their CV. The panel discussion included a linguistic validation by a comparison of the Arabic translation with the original English by assessing conceptual and content equivalence. A consensus was reached to express a concept in Arabic language to ensure that the translated version maintained content validity and produce the closest natural equivalent of the English language. In addition, we read each item aloud more than once to anticipate the possible interpretations by participants. The panel meeting lasted for 2 hours and was audio taped to have an accurate record of the discussion and to allow reviewing the items of the CAS discussed.

### Focus groups discussion of the CAS

Two focus groups were held with women who were contacted through a pre-existing social group for Saudi women in London. This was done to further refine the CAS and ensure its acceptability to participants, in terms of its format, layout, content, and wordings.

The CAS was posted, in Arabic and English, to the focus groups members one week before the discussion date. These women had agreed to complete the CAS themselves and articulated their views on its content and layout. The focus groups were held at a time and place that were convenient to the women. A topic guide was developed to guide and facilitate the discussion (Appendix S2 in File S1) and to help them reflecting on the translation of CAS, but allowing flexibility to pursue unanticipated issues.

The topic guide covered issues of: translation and language used in the questionnaire, women’s reaction to the questionnaire generally, time taken to complete it, wording, content and lay out of questions.

#### Selection of participants of the focus groups

Participants in the focus groups were purposively sampled based on the needs of refining the CAS and the quality of the initial translation. This allowed us to bring together diverse groups, in terms of their age and expertise relating to the field of translation in general and the violence against women, in particular, to maximize exploration of different perspectives and views on the CAS. To ensure the confidentiality of our participants, we provided a general description of their characteristics.

In one of the meetings of the social group, S A explained the purpose of the study and asked for volunteers. Women who agreed to participate were asked to send their CV, in order to purposively sample them for the focus group discussions.

Twenty women responded by e-mail with their CVs. Ten of them were purposively selected based on their expertise and potential to provide different community categories (employed, non-employed), different age groups, with potential different perspectives and insights on the language, wording and lay out of the CAS.

They were assigned into two focus groups, of which, four of them attended the first group (professional workers) while six were in the second group (house wives). Participants were invited by e-mail and were followed by phone call to ensure they had received this e-mail. They were e-mailed with an information sheet explaining the study purpose and their role in the discussion (Appendix S3 in File S1). The participants’ ages ranged from 35 to 45 years.

#### Conducting the focus groups

Participants were sent a copy of the original CAS and asked to complete before the focus group discussions to allow then have plenty of time to consider the measure and list their comments. However, they were not requested to hand in their completed questionnaires, but to feedback and comment on the questionnaire itself. The two focus groups took place between November and December 2008. All participants were given a £10 gift voucher as a sign of appreciation for their contribution to the discussion.

Discussions were audio taped in conjunction with written notes taking by the facilitator. These notes were used as supplementary to the tape recordings and as a backup. Thereafter, the tape recording of each focus group was listened to and then developed anonymous abridged transcript of the relevant and useful elements regarding the questions.

Analysis of focus groups involved listening to the tapes, transcribing them, reading and re-reading the transcripts and the notes. Then the necessary changes were made as suggested by the groups (Table S1 in File S1). When there were disagreements between members, the facilitator encouraged the group to discuss the inconsistencies in order to reach to an agreement between them. Additionally, disagreements were used to encourage members to elucidate their point of view and to clarify why they thought as they do. The refined questionnaire was then e-mailed to the focus groups members to double check the accuracy of changes suggested by them. However, there were no further changes suggested by the groups.

### Back-translation and comparison with the original version of CAS

A professional female bilingual translator was approached to perform the back-translation of CAS. The back translator was not aware of the intent and concepts underlying the original questionnaire. Hence, she was free of biases and expectations, in order to allow her to reveal any possible unexpected meanings or interpretations in the final version of CAS.

Finally, S A compared the back-translated version with the original English CAS version, to resolve any discrepancies or variations between the translations by referring back and forth between the original CAS and the back-translated one.

### Ethical consideration

This study was approved by the ethics committee in the Faculty of Medicine and Dentistry (University of Bristol, UK). Confidentiality of the tape recordings of the discussion and transcripts were assured and signed consent was obtained before commencing the discussion (Appendix S4 in File S1).

Participants were ensured that their comments would be anonymous and that their names would not appear on any report or publication.

The nature of the questionnaire was of a sensitive nature, and there was a possibility that some of the participants in the focus group discussions had experienced IPV and might develop emotional reaction, a professional counsellor was arranged in advance.

## Results

### Principal researcher forward translation of CAS

The initial translated version of the CAS was completed. Translation took about three hours. S A thought that question number 25 in the CAS, which asked women about the use of objects in the vagina, should be removed because it was so intrusive and was not preceded by an introductory question about the sexual relationship between partners in general. However, it was not remove at this stage and preferred to leave it for the judgments of the experts’ panel and the focus groups.

### Expert Panel translation of CAS

In addition to the linguistic considerations, the expert panel emphasized that there are psychological considerations, which place the questionnaire in a broader cultural context. For example, the wording of item one in the CAS questionnaire, intimate relationship had to be changed to be married or engaged. They thought that such modifications were required in order to deal with the emotional effect that could be created if the source language words were used. In the source language, the word intimate relationship could be offensive and may be insulting to Saudi women since it is generally not acceptable in their culture for women to have relationships outside the marriage. In Arabic, the appropriate term was thought to be “Shareek” as pronounced in Arabic, which denotes both meanings of a husband and/or a partner, which is characteristic of an intimate relationship. However, their comments were considered and discussed in the focus groups meeting, without changing the initial translation. This was done in order to be inclusive and to avoid any bias that could influence the process of translation, and comments on the questionnaire in the next stage of translation (focus groups). The panel suggested deleting question number 25 of CAS, for the same reason mentioned above.

### Focus groups discussion of the questionnaire

The focus group discussion lasted 90-120 minutes. The majority of the members of the focus groups emphasized the importance of putting spaces between sections of the questionnaire or lines, to ensure the respondents know when each section ended. The initial translated texts of the CAS were written without diacritical marks and hence, the focus groups addressed their importance. Diacritical marks, which include accent marks, tildes, and other notations, help to distinguish one letter from another and aid in pronunciation. When added or removed, they can completely change the meaning of a word or sentence.

The majority of participants noted that the questionnaire was of acceptable length and took approximately 10-15 minutes to complete. Only one woman from the second focus group filled the survey in 30 minutes.

Focus groups advised to use shorter instruction to ensure women read them completely to answer the questions. The groups agreed that the wording should be in simple Arabic forms that could be understood by women with any educational level. They also, ensured use of vowel (dialect) marks to clarify the exact meaning.

Focus groups suggested using a broad term to address intimate partnership while preserving the exact meaning of adult intimate relationship. However, they strongly recommended not to use boy or girl friends, as this is was not in Saudi culture in an explicit manner in the current time acceptable (Saudi women are not allowed to have intimate partner relationship without marriage).

Another point discussed among the groups was the ordering of the words. In English, ordering of a sentence is usually in the form of subject first, then verb (SVO). However, in Arabic, it is the other way round; verb then subject (VSO). Also Arabic adjectives typically follow the nouns, while in English; adjectives can either precede or follow depending on the adjective phrase length. For example, *a beautiful woman* is translated as إمرأ**ة** جميل**ة** *imraha jamilaha* ‘woman beautiful’. Therefore, this resulted in a re-ordering of the source sentences (English) to assimilate the word order of the target (Arabic) language for some questions.

Regarding question 25, both groups’ members suggested removing this question from CAS for the same reasons discussed above.

Thereafter, the CAS was refined in light of the comments and suggestions made by the groups’ members. However, to ensure the accuracy of the translation, all members were e-mailed with the refined version of the questionnaire for double-checking the suggested changes. There were no major corrections, apart from minor linguistic corrections to a few questions.

### Back translation of CAS

Finally, the back-translated CAS showed almost similar wording and language of the original version. However, the words ‘harassed’ me in question 13 and 16 of the original CAS, were back translated in question13 as ‘harassed me’, and in question 16 as ‘threatened me’. This was translated as such because the Arabic wordings used in these two questions were translated wrongly into different meanings in Arabic language (harassed in question 13 and threatened in question 16). This resulted in amplification in the back translation and therefore, revealed the above noticed failure to adapt to the target context and ambiguity in the source version was uncovered. This was the only noticed discrepancy between the original CAS and the back-translated one.

## Discussion

Translation is not a single process leading from a starting point ST= source text to a target point TT=target text, but a circular, basically recursive process comprising an indefinite number of feedback loops, in which it is possible and even advisable to return to earlier stages of the analysis [22]. In our study, we used different feedback loops. The expert panel ‘discussion in the process of translation of the CAS questionnaire improved the quality of translation by their critical feedback when discussing the appropriate translated words with psychological, cultural, and religious sensitivity. The panel’s feedback also had the strength of the synergistic effort between the bilingual members, especially when one of them had experience in dealing with IPV cases (psychiatrist) complemented by a second lay person who had a broad understanding in the field of IPV in similar socio-cultural communities (participated in voluntary work dealing with women exposed to IPV). Translation of CAS involved not just two languages, but a transfer from culture to another. Cultural problems often posed a great difficulty than did the linguistic problems. Hence, some texts were easy to translate, others were difficult, for example, *Have you ever been in an adult relationship*? This question could be asked directly to women of Western culture without misinterpretation. However, if used with women from Arabic background and with certain religious beliefs, as in Muslims, it would not be acceptable as such and should be phrased into a more acceptable wording, like saying: *Have you been married before*? In these cultures, it is not acceptable for a woman to have any kind of adult relationship apart from her socially or legally recognized husband. Therefore, words denoting social or ethical ideas or concepts usually have different meanings in different languages. These words needed to be understood as they are aimed to be given in the source language (e.g. partner) and conveyed them in the target language (husband in Arabic).

Our study has important strengths. The multi-method approach in translating the CAS was one of its strengths. It helped in minimising possible sources of method bias in the translation and adaptation process of the questionnaire. This was in accordance to the International translation Commission (ITC) guidelines [21]. The guidelines recommend assessment of the cultural distance between the source and target language and cultural groups in order to reduce the effect of cultural differences that are not relevant or important to the main purpose of the study. Assessment of cultural distance included considerations of differences in language, family structure, religion, lifestyle, and values [23]. We achieved this by selecting members of the focus groups who were familiar with the Arabic language’s culture and its diversity. Focus group’s members were bilingual and well oriented in the Western culture. Additionally, three had expertise in linguistics or psycholinguistics, which provided a valid contribution to the quality of the CAS translation and adaption of the survey questionnaire.

ITC Guidelines also recommend that specified qualifications beyond knowledge of the two languages are essential in the process of translation. Knowledge of the cultures, and at least general knowledge of the subject matter and its principles, was part of the selection criteria for the expert panel, the members of the focus groups, and the independent back translator. This was done using their CVs and their level of experiences in the field of IPV. The use of these multiple methods (expert panel, focus groups, and back-translation) with participants experienced in linguistics and psychology fields ensured a high-quality translation of the CAS.

Furthermore, ITC Guidelines pointed to the importance of preserving both the linguistic as well as the connotative meaning in the translation of words. A good linguistic translation preserves meaning, but to preserve the connotative meaning, a target language word that preserves the “emotion” associated with the word is necessary. This was observed in question number 1 in the CAS “have you been in an intimate relationship?” This implies the notion of being in an intimate relationship. The expert panel and focus groups attempted to use a proper Arabic word (Shareek) that preserved the meaning, and the emotion of the translated word as in the CAS questionnaire, which increased the confidence that items would be understood by the questionnaire respondents and consistent with the original meaning of the items.

This study has also some limitations. One of the limitations is that the translation was done via Saudi women in the UK, and Saudi population living in the UK might be a self-selected group. Furthermore, removing question 25 from the CAS might affect the comparability of our survey with other studies that used CAS. However, studies conducted elsewhere using CAS had deleted the same question for similar reasons (personal communication).

Another limitation was that the cultural adaptation and translation procedure carried out in this study was focused on Saudi women. This was in line with our need to develop the Arabic version of the CAS to investigate the prevalence of IPV for this particular population. Therefore this particular translation may not be applicable to other groups, and would need to be reviewed prior to generalised use. Further validation of the Arabic version of the CAS is recommended to ensure the linguistic, conceptual and cultural equivalence between the original and the translated version of the CAS in Saudi population and similar Arabic speaking countries.

## Implications

CAS translation was specifically geared towards the Saudi population; however, it has potential to be used in other Arabic populations. The Arabic version of the CAS will help to explore the problem of IPV among Saudi women and possibly other Arabic-speaking women. This is important, if it will be used to document follow up in longitudinal studies or intervention studies among abused women, as well as in cross-sectional study to measure the prevalence and incidence of IPV. In addition, it allows a comparison of the results of studies from different cultures.

## Supporting Information

File S1 **Supporting information: Appendix S1, Appendix S2, Appendix S3, Appendix S4, and Table S1.**
(DOCX)
