# Supplementary material for: English to Arabic Translation of the Composite Abuse Scale (CAS): A Multi-Method Approach
Source: PLoS One. 2013 Sep 25;8(9):e75244. doi: 10.1371/journal.pone.0075244 (PMC3783446; doi:10.1371/journal.pone.0075244)
Supplement: File S1 — Supporting information: Appendix S1, Appendix S2, Appendix S3, Appendix S4, and Table S1. (DOCX) [file pone.0075244.s001.docx]

Appendix S1: Survey questionnaire in English

ID Number:


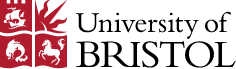


Faculty of Medicine and Dentistry

Academic Unit of Primary Health Care

Bristol 25 Belgrave Road, BS8 2AA

**Tel:** **01173313817**

Email: samia.alhabib@bristol.ac.uk

The Health of Saudi Women Living in the UK

Please complete this questionnaire and take the time to read each question carefully. Please read the information sheet provided before commencing to answer the questions. Try to answer all the questions as honestly as you can. Please ***circle*** the answer that is best for you. There is no need to write your name on the questionnaire. Everything you say will be treated as confidential.

Please return the completed questionnaire in the pre-paid envelope provided within 2 weeks.

With many thanks for your contribution.

**General Health and well-being**

This section asks about your general health. Please answer all the questions. If unsure about which response to give to a question, please choose the one that appears most appropriate. This can often be your first response. Please ***circle*** the answer that is best for you.

**A1**. In general, would you say your health is?

| 1. Excellent 2.Very good 3. Good 4. Fair 5.Poor |
| --- |

**A2. Compared to one year ago**, how would you rate your health in general **now**?

| 1 | Much better now than one year ago |
| --- | --- |
| 2 | Somewhat better now than one year ago |
| 3 | About the same |
| 4 | Somewhat worse now than one year ago |
| 5 | Much worse now than one year ago |

The following items are about activities you might do during a typical day. Does **your health now limit you** in these activities? If so, how much?

**A3. Vigorous activities**, such as running, lifting heavy objects, participating in strenuous sports

| **1.** Yes, limited a lot | **2.** Yes, limited a little | **3.** No, not limited at all |
| --- | --- | --- |

**A4.** **Moderate activities**, such as moving a table, pushing a vacuum cleaner, bowling, or playing golf

| **1.** Yes, limited a lot | **2.** Yes, limited a little | **3.** No, not limited at all |
| --- | --- | --- |

**A5.** Lifting or carrying groceries

| **1.** Yes, limited a lot | **2.** Yes, limited a little | **3.** No, not limited at all |
| --- | --- | --- |

**A6.** Climbing **several** flights of stairs

| **1.** Yes, limited a lot | **2.** Yes, limited a little | **3.** No, not limited at all |
| --- | --- | --- |

**A7.** Climbing **one** flight of stairs

| **1.** Yes, limited a lot | **2.** Yes, limited a little | **3.** No, not limited at all |
| --- | --- | --- |

**A8.** Bending, kneeling, or stooping

| **1.** Yes, limited a lot | **2.** Yes, limited a little | **3.** No, not limited at all |
| --- | --- | --- |

A9. Walking more than a mile

| **1.** Yes, limited a lot | **2.** Yes, limited a little | **3.** No, not limited at all |
| --- | --- | --- |

A10. Walking several blocks

| **1.** Yes, limited a lot | **2.** Yes, limited a little | **3.** No, not limited at all |
| --- | --- | --- |

A11. Walking one block

| **1.** Yes, limited a lot | **2.** Yes, limited a little | **3.** No, not limited at all |
| --- | --- | --- |

**A12.** Bathing or dressing yourself

| **1.** Yes, limited a lot | **2.** Yes, limited a little | **3.** No, not limited at all |
| --- | --- | --- |

During the **past 4 weeks**, have you had any of the following problems with your work or other regular daily activities **as a result of your physical health**?

|  | Yes | No |
| --- | --- | --- |
| **A13.** Cut down the amount of time you spent on work or other activities | 1 | 2 |
| A14. Accomplished less than you would like | 1 | 2 |
| **A15.** Were limited in the **kind** of work or other activities | 1 | 2 |
| **A16.** Had **difficulty** performing the work or other activities (for example, it took extra effort) | 1 | 2 |

During the **past 4 weeks,** have you had any of the following problems with your work or other regular daily activities **as a result of any emotional problems** (such as feeling depressed or anxious)?

|  | Yes | No |
| --- | --- | --- |
| **A17.** Cut down the **amount of time** you spent on work or other activities | 1 | 2 |
| A18. Accomplished less than you would like | 1 | 2 |
| **A19.** Didn't do work or other activities as **carefully** as usual | 1 | 2 |

**A20.** During the **past 4 weeks,** to what extent has your physical health or emotional problems interfered with your normal social activities with family, friends, neighbors, or groups?

| **1.** Not at all | **2.** Slightly | **3.** Moderately | **4.** Quite a bit | **5.** Extremely |
| --- | --- | --- | --- | --- |

**A21.** How much **bodily** pain have you had during the **past 4 weeks**?

| 1. None | 2. Very mild | 3. Mild | 4. Moderate | 5.Severe | 6.Very severe |
| --- | --- | --- | --- | --- | --- |

**A22.** During the **past 4 weeks,** how much did **pain** interfere with your normal work (including both work outside the home and housework)?

| **1.** Not at all | **2.**A little bit | **3.** Moderately | **4.** Quite a bit | **5.** Extremely |
| --- | --- | --- | --- | --- |

How TRUE or FALSE is ***each*** of the following statements for you.

|  | Definitely true | Mostly true | Don’t know | Mostly false | Definitely false |
| --- | --- | --- | --- | --- | --- |
| **A23.** I seem to get sick a little easier than other people | 1 | 2 | 3 | 4 | 5 |
| **A24.** I am as healthy as anybody I know | 1 | 2 | 3 | 4 | 5 |
| **A25.** I expect my health to get worse | 1 | 2 | 3 | 4 | 5 |
| A26. My health is excellent | 1 | 2 | 3 | 4 | 5 |

**A27.** During the **past 4 weeks,** how much of the time has your **physical health or emotional problems** interfered with your social activities (like visiting with friends, relatives, etc.)?

| **1.** All of the time | **2.** Most of the time | **3.** Some of the time | **4.** A little of the time | **5.** None of the time |
| --- | --- | --- | --- | --- |

These questions are about how you feel and how things have been with you **during the past 4 weeks**. For each question, please give the one answer that comes closest to the way you have been feeling.

How much of the time during the **past 4 weeks** . . .

|  | All of the time | Most of the time | A good bit of the time | Some of the time | A Little of the Time | None of the Time |
| --- | --- | --- | --- | --- | --- | --- |
| **A28.** Did you feel full of pep? | 1 | 2 | 3 | 4 | 5 | 6 |
| **A29.** Have you been a very nervous person? | 1 | 2 | 3 | 4 | 5 | 6 |
| **A30.** Have you felt so down in the dumps that nothing could cheer you up? | 1 | 2 | 3 | 4 | 5 | 6 |
| **A31.** Have you felt calm and peaceful? | 1 | 2 | 3 | 4 | 5 | 6 |
| **A32.** Did you have a lot of energy? | 1 | 2 | 3 | 4 | 5 | 6 |
| **A33.** Have you felt downhearted and blue? | 1 | 2 | 3 | 4 | 5 | 6 |
| **A34.** Did you feel worn out | 1 | 2 | 3 | 4 | 5 | 6 |
| **A35.** Have you been a happy person? | 1 | 2 | 3 | 4 | 5 | 6 |
| **A36.** Did you feel tired? | 1 | 2 | 3 | 4 | 5 | 6 |

**B. Domestic relationships and well-being**

This section asks about your experiences in adult intimate relationships. By adult intimate relationship, we mean a husband, partner or boy/girlfriend for longer than one month. Please circle the answer that is best for you.

**B1.** Have you ever been in an adult intimate relationship?

(Since you were 16 years of age) Yes…..1 ***Go to question B2***

No…....2 if ***no please go to the end of questionnaire***

**B2.** Are you currently in a relationship? Yes……1

No…….2 ***Go to question B4***

**B3.** Are you currently afraid of your partner? Yes…….1

No…….2

**B4**. Have you ever been afraid of any partner? Yes…….1

No……..2

We would like to know if you experienced any of the actions listed below and how often it happened during the past 12 months. If you were not with a partner in the past 12 months, could you please answer for the last partner that you had? Please circle the number, which matches the frequency over a 12-month period that it happened to you.

***How often it happened?***

| Action | Never | Only  Once | Several  Times | Once/  Month | Once/  Week | Daily |
| --- | --- | --- | --- | --- | --- | --- |
| **20.** Told me that I was crazy | 0 | 1 | 2 | 3 | 4 | 5 |
| **21.** Told me that no one would ever want me | 0 | 1 | 2 | 3 | 4 | 5 |
| **22.** Took my wallet and left me stranded | 0 | 1 | 2 | 3 | 4 | 5 |
| **23.** Hit or tried to hit me with something | 0 | 1 | 2 | 3 | 4 | 5 |
| **24.** Did not want me to socialize with my female friends | 0 | 1 | 2 | 3 | 4 | 5 |
| **25.** Refused to let me work outside the home | 0 | 1 | 2 | 3 | 4 | 5 |
| **26.** Kicked me, bit me or hit me with a fist | 0 | 1 | 2 | 3 | 4 | 5 |

How often it happened

| Action | Never | Only  Once | Several  Times | Once/  Month | Once/  Week | Daily |
| --- | --- | --- | --- | --- | --- | --- |
| **11.** Hung around outside my house | 0 | 1 | 2 | 3 | 4 | 5 |
| **12.** Blamed me for causing their violent behaviour | 0 | 1 | 2 | 3 | 4 | 5 |
| **13.** Harassed me over the telephone | 0 | 1 | 2 | 3 | 4 | 5 |
| **14.** Shook me | 0 | 1 | 2 | 3 | 4 | 5 |
| **15.** Tried to rape me | 0 | 1 | 2 | 3 | 4 | 5 |
| **16.** Harassed me at work | 0 | 1 | 2 | 3 | 4 | 5 |
| **17.** Pushed, grabbed or shoved me | 0 | 1 | 2 | 3 | 4 | 5 |
| **18.** Used a knife or gun or other weapon | 0 | 1 | 2 | 3 | 4 | 5 |
| **19.** Became upset if dinner/housework wasn’t done when they thought it should be | 0 | 1 | 2 | 3 | 4 | 5 |

How often it happened?

| Action | Never | Only  Once | Several  Times | Once/  Month | Once/  Week | Daily |
| --- | --- | --- | --- | --- | --- | --- |
| **20.** Told me that I was crazy | 0 | 1 | 2 | 3 | 4 | 5 |
| **21.** Told me that no one would ever want me | 0 | 1 | 2 | 3 | 4 | 5 |
| **22.** Took my wallet and left me stranded | 0 | 1 | 2 | 3 | 4 | 5 |
| **23.** Hit or tried to hit me with something | 0 | 1 | 2 | 3 | 4 | 5 |
| **24.** Did not want me to socialize with my female friends | 0 | 1 | 2 | 3 | 4 | 5 |
| **25.** Refused to let me work outside the home | 0 | 1 | 2 | 3 | 4 | 5 |
| **26.** Kicked me, bit me or hit me with a fist | 0 | 1 | 2 | 3 | 4 | 5 |

How often it happened?

| Action | Never | Only  Once | Several  Times | Once/  Month | Once/  Week | Daily |
| --- | --- | --- | --- | --- | --- | --- |
| **27.** Tried to convince my friends, family or children that I was crazy | 0 | 1 | 2 | 3 | 4 | 5 |
| **28.** Told me that I was stupid | 0 | 1 | 2 | 3 | 4 | 5 |
| **29.** Beat me up | 0 | 1 | 2 | 3 | 4 | 5 |

General questions

Please give your age:

Please give number of children:

Please give number of years living in UK:

Please circle your marital status: 1. Married.

2. Un-married.

If you circle one in previous question, please give us how many years you have been married:

Educational level of husband:

1. Postgraduate 2. University degree 3. Completed High school

4. Secondary school 5. Primary school 6. Illiterate

Your educational level:

1. Postgraduate 2. University degree 3. Completed High school

4. Secondary school 5. Primary school 6. Illiterate

30. Thank you for the time to answer this questionnaire. My research also entails interviews of some women to clarify issues in more detail. Women will be paid a £10 voucher for their contribution to the interview. Therefore, would you be happy to be contacted about taking part in an interview*?*

Yes…….1

No…….2

Thank you for completing this questionnaire. If you need further help, you can call a free 24-hour national helpline in England: **0808 2000 247**, Welsh 24 Help-line: **0808 80 10 800.** This will provide access to 24-hour emergency information service, including safety planning and translation facilities. Calls from landlines will not appear on the telephone bill. You can call also the NHS direct: **08454647** or contact your GP for further help. You can also call Dr. Samia Alhabib on telephone **07827669067,** or e-mail her using the following e-mail:

[Samia.alhabib@bristol.ac.uk](mailto:Samia.alhabib@bristol.ac.uk).

Appendix S2: focus groups guide

Saudi women’s health status living in the UK

Focus group

Introduction

I am Samia Alhabib, doing a PhD in University of Bristol. My project will be about the health status of Saudi women living in the UK. I believe you have received a copy of the questionnaire, information sheet, and the consent form, and had the chance to try to answer the study questionnaire. During the discussion, you will be given your views on the wording, quality of translated language, layout and contents of the questionnaire. You would also be asked to comment on the documentation that will be sent with the questionnaire. The questionnaire and the accompanying paperwork will then be refined in light of the comments made.

Today's focus group will be tape recorded and this is done so that we have a comprehensive record of all of the issues that we discuss today. I would like to assure everyone that all your responses are confidential. By this I mean that only the research team will see the transcriptions of the tape recordings and that when the final report of all the focus groups is written no one will be identified individually. Is everyone ok with that?

Ground rules

- There is no right or wrong answer.

- All views are equally valid.

- Ask people to speak one at a time if possible.

- I am here to facilitate the discussion and that I am happy for participants to question one another and discuss issues among themselves and not simply to me.

- Do not use each other names during the discussion.

Appendix S3: Focus groups’ information sheet


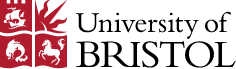


Faculty of Medicine and Dentistry

Academic Unit of Primary Health Care

Bristol25 Belgrave Road, BS8 2AA

Tel: 01173313817

Email:samia.alhabib@bristol.ac.uk **samia.alhabib@bristol.ac.uk**

Information sheet B

Version 1, 30 May 2008

The Health of Saudi Women Living in the UK

Thank you for considering taking part in this research. This information sheet provides details about the study. Please take time to read it before deciding whether or not you would like to take part.

The aim of the study:

This research will be carried out by researchers at Bristol University and funded by the Saudi Cultural bureau. This study aims to assess the health of Saudi women living in the UK. In this study, health refers to physical and psychological aspects of women’s well-being.

Saudi women living in the UK will be posted a questionnaire to complete and return to the researcher. The questionnaires will focus on your general health, well-being, and any experience of physical and/or psychological distress at some point in your life.

The aim of the discussion group

It is very important that women being sent the questionnaire find it acceptable, clear and understand the questions being asked. The purpose of the discussion group is to ensure that this happens.

If you took part in the discussion group, you would be asked to complete a questionnaire and then, during the discussion group, to give your views on its content and layout. You would also be asked to comment on the documentation that will be sent with the questionnaire. The questionnaire and the accompanying paperwork will then be refined in light of the comments made.

The group will be held at a time and place that is convenient to those taking part. The group discussion will last about 2 hours and will be audio-taped.

Why have I been chosen?

You have been asked to take part in a discussion group because you are a Saudi woman living in the UK for more than six months. The questionnaire is to be posted to Saudi women who have lived in the UK for more than six months.

Do I have to take part?

You do not have to take part in the group discussion. If you choose to take part, you can refuse to answer a question if you wish and can withdraw from the group at any time without giving a reason and your legal rights being affected.

Will my taking part in this study be kept confidential?

All the information collected will be treated as confidential and stored securely at Bristol University. The only people who will see the information you provide will be the researcher and her two supervisors.

What will happen to the results of the study?

Everyone who has taken part can ask to be posted a summary of the findings at the end of the study. Findings from the study will be written up for publication in scientific journals and presented at international conferences. No one will be able to identify any of the participants from the published findings. Once the study has ended, all information provided by those who have taken part will be destroyed.

What I have to do now?

If you are happy to take part in the group discussion, please sign the consent form attached.

Contact for further information:

If you would like to talk to the researcher, **Samia Alhabib**, please call her on telephone number **07827669067.** Samia speaks both English and Arabic.

Appendix S4: Consent form

Title of project: Saudi women’s Health Status living in the UK.

Name of Researcher: **Dr. Samia Alhabib**

I have read and understood the information sheet for (version 1, 30 April 2008) the above study. □

I have had the opportunity to consider the information, ask questions and have had these answered satisfactorily. □

I understand that my participation is voluntary and that I am free to withdraw at any time without giving reason, without my medical care or legal rights being affected. □

I understand that the researchers may look at relevant sections of my data collected during the study, where it is relevant to my taking part in this research. I give permission for these individuals to have access to my data. □

I agree to take part in the above study. □

Name of Participant: Name of person taking consent:

Date: Date:

Signature: Signature:

Table S1: Summary of the changes and decisions made to the whole questionnaire survey that resulted from the focus groups.

| Suggested Changes | Decisions made | Source |
| --- | --- | --- |
| General changes | | |
| Should put spaces between sections. | Spaces put between sections to indicate more clearly that a section had ended. | Focus groups |
| Numbers should be in Arabic. | Numbers put in Arabic so that they are consistent with the Arabic wording | Focus groups |
| Short vowels marks must be used. | Short vowels added because without them a word may have a completely different meaning, e.g. followed me, could be translated in Arabic to; يتبعني. Without vowels marks, this could mean follow my orders, but with vowels marks, َيتبَعٌني, it will give the right meaning, i.e. followed me in order to spy on me and know my movements. | Focus groups |
| Suggested alternative translated Arabic words, in most of the questions, which were slightly different from the initial translation, but they give a clear meaning. | Alternative Arabic words were made in many occasions, when consensus was reached between members of the focus groups, e.g. threw me, my initial translation was; دفعني, which means just push me.  To make it more descriptive, groups suggested more elaborative words; طرحني أرضاً, which means push me until I felt down. | Focus groups |
| Instructions for sections or questions should be written in bold. | Instructions in sections and questions were written in bold in order to make it clear for the participants. | Focus groups |
| Pointed to a few Arabic spelling mistakes, and use of Arabic commas. | Spelling checked and commas were inserted. | Focus groups and expert panel |
| To lighten the shading of questions. | Shading was lightened, because it looks more convenient and clearer to the reader | Focus groups |
| Put the age in categories. | Ages were not put in categories, because we felt it would be better to treat age as a continuous variable. | Focus groups |
| Marital status question should married and married | Not to change, because we need to consider the different marital status in relation to other variables in the analysis. Thus, the choices stayed as; married, never married, and divorced. | Focus groups |
| In Q31: Asking women if they agree to be interviewed with the principal researcher and where they will be given imbursements for taking part? The suggestion is to give vouchers instead of paying transportation. | Agreed to give vouchers, because of some logistic problem, as processing the payment to participants via the University and this might breach the confidentiality, especially that names and address should be handled to the University in order to complete the payment to participating women. In addition to the fact that we do not know where will be the interview held, because it will be determined by the interviewee. | Focus groups |
| To add the principle researcher’s e-mail | E-mail was added, because some women feel shy about discussing sensitive issues over the telephone. | Focus groups |
| To reduce the choices when answering some questions. | Choices were not changed, because reducing them might affect the internal validity of the questionnaire. | Focus groups |
| Changes made to SF-36 Survey | | |
| Use Kilometres instead of miles. | Miles were converted into kilometers but miles were stated in brackets, as some women may still think in terms of miles rather than kilometers. | Focus groups |
| Suggested to use time of walking than distance. | Distance was left unchanged, because changing to time would change the meaning of the original question. | Focus groups |
| Changes made to CAS | | |
| To delete boy/girl friends in the first section of CAS | Alternative Arabic word was used that include whether women were married, engaged, or separated (partner=شريكك), in light of respecting the Saudi cultural values and norms. | Expert panel and focus groups |
| To carefully select the Arabic meaning of partner. | Agreed on proper Arabic word. Other researchers used husband, but we used a word that explain intimate adult relationship (شريكك) = partner, that does not necessarily imply the legality of the partnership status, but include all possibilities of relationships without insulting the Saudi culture. | Focus groups and expert panel |
| To delete question 25, which asked about putting foreign objects in the vagina? | Q 25 was deleted, because it is not acceptable and deeply offensive in Saudi culture. | Focus groups and expert panel |
| Changes made to information sheet | | |
| Not to mention the Embassy in the information sheet. | The word Embassy was removed, as we understand that some women may feel uncomfortable or even unsafe answering a questionnaire associated with the Embassy because we need women to feel safe and complete the questionnaire without fears. However we agreed to re-phrase it to Ethical committee. | Focus groups |
| To mention how generally the Saudi women population will benefit from this project in the information sheet, e.g. prevention or awareness programs. | General aims added, as suggested, in the information sheet, because this will encourage women to take part. | Focus groups |
